# Participatory Planning, Monitoring and Evaluation of Multi-Stakeholder Platforms in Integrated Landscape Initiatives

**DOI:** 10.1007/s00267-017-0847-y

**Published:** 2017-03-21

**Authors:** Koen Kusters, Louise Buck, Maartje de Graaf, Peter Minang, Cora van Oosten, Roderick Zagt

**Affiliations:** 1 Tropenbos International, Wageningen, Netherlands; 2 EcoAgriculture Partners, Washington DC, USA; 3 Cornell University, Ithaca, USA; 4 World Agroforestry Centre, Nairobi, Kenya; 5 Centre for Development Innovation, Wageningen University and Research, Wageningen, Netherlands

**Keywords:** Landscape approach, Multi-stakeholder platform, Planning, Monitoring, Evaluation

## Abstract

Integrated landscape initiatives typically aim to strengthen landscape governance by developing and facilitating multi-stakeholder platforms. These are institutional coordination mechanisms that enable discussions, negotiations, and joint planning between stakeholders from various sectors in a given landscape. Multi-stakeholder platforms tend to involve complex processes with diverse actors, whose objectives and focus may be subjected to periodic re-evaluation, revision or reform. In this article we propose a participatory method to aid planning, monitoring, and evaluation of such platforms, and we report on experiences from piloting the method in Ghana and Indonesia. The method is comprised of three components. The first can be used to look ahead, identifying priorities for future multi-stakeholder collaboration in the landscape. It is based on the identification of four aspirations that are common across multi-stakeholder platforms in integrated landscape initiatives. The second can be used to look inward. It focuses on the processes within an existing multi-stakeholder platform in order to identify areas for possible improvement. The third can be used to look back, identifying the main outcomes of an existing platform and comparing them to the original objectives. The three components can be implemented together or separately. They can be used to inform planning and adaptive management of the platform, as well as to demonstrate performance and inform the design of new interventions.

## Introduction

The landscape approach stresses that conservation and development challenges are best addressed at the level of the landscape, as this is where synergies between ecosystem health and livelihood security can be realized. The approach specifies the need for iterative processes of understanding, negotiation, and decision-making among actors from different sectors (Scherr et al. 2013; Sayer et al. 2013; see Reed et al. 2016 for an extensive review of the literature). The focus on collaborative planning and management corresponds with notions of cross-sectoral forms of environmental governance (e.g., Lemos and Agrawal 2006).

The rising popularity of the landscape approach has resulted in growing investments in so-called integrated landscape initiatives (ILIs) (Milder et al. 2014; Estrada-Carmona et al. 2014, Hart et al. 2015). These initiatives typically have multiple objectives, cover multiple landscape components (e.g., agricultural fields, natural forest areas, tree plantations, fallows, riparian areas, rivers, and settlements), and are built around multi-stakeholder processes. A key component of many ILIs is the support of a multi-stakeholder platform to facilitate the ‘co-design’ of the landscape by public, private, and civil society actors (ibid.). Platforms provide a space in which stakeholders can share and discuss their interests. Defined broadly, they include various forms of organized multi-stakeholder collaboration, including coalitions, partnerships, and management boards. The platform is meant to support the joint identification of options to balance the various interests that may exist in the landscape; long-term and short-term, local up to global, public, private and civic (Zagt and Chavez-Tafur 2014; Kozar et al. 2014). By providing a space for discussion, negotiation and decision-making, a multi-stakeholder platform is expected to strengthen the landscape governance system, which can be conceived as the function of different formal, semi-formal and informal decision-making processes that together shape the landscape (see, e.g., Görg 2007; Van Oosten 2013; Salvemini and Remple 2014). Although a multi-stakeholder platform may not cover the entire governance system, it is expected to function as an institutional coordination mechanism helping to align and integrate planning and decision-making processes of different actors.

With growing interest in multi-stakeholder platforms as part of ILIs, there is a need for adequate methods to aid their planning, monitoring, and evaluation (PME), with an emphasis on learning. This provides several challenges. Multi-stakeholder platforms tend to be places of ambiguity, uncertainty, and complexity. Considering the different interests within a platform, stakeholders will likely have different perspectives on optimal outcomes. Moreover, objectives may be revised periodically, based on evolving understanding of the landscape situation and processes of negotiation. We therefore argue that there is a need for PME methods that can deal with this complexity, while being practical and affordable. Moreover, we postulate that such PME methods would need to pay specific attention to the features and quality of the multi-stakeholder process itself, as this is expected to affect the overall effectiveness of the platform (Buck et al. 2006; Kusters 2015a; Minang et al. 2015).

In this article we propose a general framework for such a method. It can be used to guide participatory assessments in order to generate relevant information for planning, adaptive management, and evaluation. The method we propose offers platform members (and organizations that are investing in the platform) a structure for reflecting on strategies, processes, and performance. Issues of efficiency and impact are outside the scope of this method, as these require long-term monitoring and evaluation approaches that include baselines or counterfactuals. Below, we will first outline some of the main characteristics of multi-stakeholder platforms and associated challenges, followed by a short description of the development and piloting of our method. We then introduce the three questions that form the core of the method and elaborate on the method’s implementation through multi-stakeholder workshops, including a summary of the results of the two cases where we tested the method. In the final section we reflect on some conditions for successful implementation (which may apply to participatory workshops in general), and possible uses of the proposed method.

### Multi-Stakeholder Platforms: Characteristics and Challenges

Interest in multi-stakeholder platforms for natural resource management is not new (see, e.g., Edmunds and Wollenberg 2002; Warner et al. 2002). In agricultural development, experimentation with so-called innovation platforms has focused on agricultural technologies and practices (see, e.g., Nederlof and Pyburn 2012; Klerkx et al. 2013; Sanyang et al. 2014; Ros-Tonen et al. 2015a), and their performance has been researched (e.g., Pamuk et al. 2014). Multi-stakeholder platforms in the context of ILIs tend to be more heterogeneous than such agricultural innovation platforms, which means that interests are more likely to be opposing. Moreover, their objectives do not relate to just one sector, but explicitly include multiple goals, covering different sectors. In the context of ILIs multi-stakeholder platforms are often established to provide a permanent space for discussions related to the use of natural resources in a certain area. Some of these platforms may be designed as formal government entities, while others operate as informal and flexible networks. Many have an external initiator, while others grow spontaneously from local initiatives (Kozar et al. 2014). A platform may have context-specific objectives that are defined at the onset (e.g., addressing the loss of soil fertility in the area while also improving productivity), but these may be adjusted by the platform over time, as the result of negotiations and discussions among its members. Considering their inherent complexity, multi-stakeholder platforms typically entail processes of ‘muddling through’ (Sayer et al. 2008, see also Lindblom 1959). Bridging organizations–often NGOs or research institutions–can play a crucial role as facilitators, creating linkages between the various actors, and supporting negotiations, collective learning and conflict resolution (Ros-Tonen et al. 2014; 2015b; see also Clark et al. 2011).

Developing effective and sustainable multi-stakeholder platforms is complex for several reasons. First, the transaction costs are high, as multi-stakeholder processes require large time investments from the participants, while the immediate benefits are not always clear or well-defined. This can discourage some actors to participate – the private sector in particular (Hart et al. 2015). Related to that, the continued existence of a platform will often be contingent upon the long-term commitment of an institutional host and skilled facilitators (Sayer et al. 2016a). Second, many multi-stakeholder arrangements lie outside formal democratic structures, and may operate under context-specific rules, without checks and balances. This raises questions about accountability and representation (Zagt and Chavez-Tafur 2014). Third, there may be large differences in power between the various actors with an interest in the landscape (and within stakeholder groups), which are likely to influence the outcomes of the process. A major challenge is thus to ensure the active participation of all actors, without discussions becoming dominated by the most powerful (Kozar et al. 2014). Fourth, multi-stakeholder platforms may be established based on certain management interests, such as the control of erosion and water usage in a watershed area, or sustainable land use and biodiversity conservation in an agricultural frontier area. However, the socio-ecological boundaries that inform the formation of multi-stakeholder platforms seldom coincide with existing administrative boundaries that form the basis of spatial decision-making by decentralized governments (Van Oosten 2013). This complicates the process of translating outcomes of landscape-level multi-stakeholder processes into actual policies and regulations (Kusters 2015b). Last but not least, challenges in the landscape are often urgent and wicked, and just getting stakeholders around a table will not automatically result in changes on the ground (Balint et al. 2011). Indeed, there is a risk that platforms turn into talking groups with little tangible result. Challenges such as the ones described above underline the need for practical tools and guidelines to help with the planning of collaborative action, adaptive management, and evaluation of platforms.

### Design Principles and Method Development

We set out to develop a practical and cost-effective method for PME of multi-stakeholder platforms in ILIs that can be used in a workshop that involves the (potential) members of the platform. While designing the method we used the following principles:

- User focus: The end-users should be kept in mind at all times, as described in Patton’s (2008) utilization-focused evaluation. For our method this means that it must be useful for the platform and its individual members. It should stimulate collective learning, by offering an opportunity to exchange experiences and views, and by jointly identifying options for the future. In addition, the method should be useful for the organizations that support or facilitate the multi-stakeholder platform, which can include conservation or development organizations, bridging organizations, companies, and government agencies. They should be able to use the method to improve their planning, management, and evaluation practices.
- Pragmatism: All too often metrics sets are cumbersome in size, and measurement becomes too time consuming and costly to be practical. Our aim was therefore to select an easy-to-use minimal set of criteria–comprehensive enough to be credible, yet simple enough to be practical. Also, as projects often have little budget available for monitoring and evaluation, we strived for a method that is relatively inexpensive and can be completed in 1 to 3 days, either by external evaluators or platform members themselves.
- Participation: The role of external advisors or consultants claiming authoritative expertise in PME is problematic, especially when they do not account for the views of stakeholders (Arts and Goverde 2006). We opted for a participatory approach as much as possible, where stakeholders help generate information flows in all directions. Involving different stakeholders helps to uncover diverse views and lead to discussions and better understanding of the issues among the participants. Moreover, people are more likely to use the results if they have been part of the assessment process (Patton 2008).
- Value judgment: Parts of the method rely on value judgments of stakeholder representatives regarding the platform’s performance. Their knowledge and perceptions are considered a rich source of data. However, value judgments are prone to bias, so it is crucial to obtain the best representation possible of stakeholders in the assessment, and to triangulate results (FAO 2011, Kusters et al. 2011). Another measure to help overcome bias and ensure the validity of the data is to include multiple members of respective stakeholder groups in the assessment.

We developed the method in an iterative manner. A literature review resulted in a framework of aspirations that are common across ILIs, and a list of principles and associated criteria that can be used to assess the quality of multi-stakeholder processes. These were then discussed, revised and refined by the authors, with input from other experts representing a range of disciplines and organizations. This resulted in a first version, which was tested during two pilot workshops. First, we organized a full-day workshop with 15 members of an existing multi-stakeholder platform in East Kalimantan, Indonesia. Here the focus was on the participatory assessment of the platform’s internal process and outcomes. Second, we conducted a workshop with 45 stakeholder representatives in the Juabeso-Bia landscape in Western Region, Ghana, where the focus was on participatory planning. Based on the two pilots the method was further revised and refined.

### Looking Ahead, Inward, and Back

Our approach to participatory PME of multi-stakeholder platforms involves three components: looking ahead, inward, and back. Looking ahead refers to the joint identification of priorities for multi-stakeholder collaboration in a given landscape. It can function as a starting point for strategic planning of new or renewed collaboration between stakeholders. By looking inward, the members of an existing platform can assess the quality of the multi-stakeholder process within the platform and identify ways to improve how the platform operates internally. By looking back, platform members can compare the outcomes of the platform to its original objectives and reflect on its performance. This will generate information that can be used by the platform (and organizations that invest in it) to report on the outcomes of their work, while also generating lessons to improve the platform’s effectiveness in the future. Corresponding to the three objectives we distinguish between three main PME questions:

1. Looking ahead: What are the priorities for collaboration in the future?
2. Looking inward: What is the quality of the multi-stakeholder process within the platform?
3. Looking back: To what extent has the platform met its objectives?

The three questions can be addressed together or separately, depending on the learning needs of the participating people and organizations. Below we address each question in turn.

#### Looking ahead: what are the priorities for collaboration in the future?

The Landscape Measures Framework developed by EcoAgriculture Partners and Cornell University distinguishes between four sustainable landscape goals that are typically pursued by ILIs. These goals relate to conservation, production, livelihoods, and institutional strengthening (Buck et al. 2006). In the context of an ILI, a multi-stakeholder platform intends to function as an institutional mechanism for achieving these goals. Ideally a platform engenders collaboration between stakeholders with different interests and levels of influence throughout a landscape, which could, for example, lead to resource mobilization, collaborative research and monitoring, promotion of synergistic land use practices, increased participation in decision-making and planning, the identification of market opportunities and policy barrier removal (Denier et al. 2015). A variety of tools have been developed to help facilitate this collaborative planning and partnership development process (Buck et al. 2017). Although the exact role and aspirations of a multi-stakeholder platform will vary from place to place, we distinguish between four general aspirational outcomes–aspirations in short (after Scherr et al. 2013):

- Shared long-term goals and action plan: In a platform different stakeholders can share their ideas about the future of the landscape, discuss what are the common interests, address potential areas of conflict, and identify shared long-term goals. When common goals have been defined, they can be translated into a joint medium-term or short-term action-plan for the landscape, outlining practical steps toward the long-term goals.
- Practices and policies advance conservation, livelihood and production objectives: Through collaboration stakeholders can jointly identify options to optimize synergies between production practices, livelihoods and the conservation of biodiversity and environmental services. For example through the promotion of agroforestry, eco-labeling, and sustainable supply chain development. Likewise, through collaboration stakeholders can identify options to align conservation practices with the interests of other stakeholders in the landscape, e.g., through schemes for compensation, payments for environmental services, developing ecotourism, etc.
- Improved monitoring and land-use planning: A multi-stakeholder platform can provide the basis for collaborative monitoring and planning processes. Dedicated monitoring efforts are necessary to track developments in the landscape (including land-cover changes, land-use practices, policies and investments) and will be more effective when stakeholders work together, combining scientific and participatory methods. The results of collaborative monitoring, in turn, can inform adaptive land-use planning processes. Ideally, land-use planning is a collaborative process as well, allowing the full participation of all relevant stakeholders and making optimal use of local and scientific knowledge, in order to inform both public and private decision-making.
- Responsive institutions: Public, private, civic, and academic actors often operate in silos. Even within the civic sector, different organizations active in the same landscape may hardly know of each other’s activities, which can lead to inefficiency and even conflict. Collaboration in a platform allows stakeholders to align and harmonize their policies and practices. A multi-stakeholder platform offers the space to share ideas and suggestions, which increases the chance that stakeholders will actually use the input from others in adaptive planning and decision-making.

To capture these aspirations in our proposed methodology, we specify two criteria for each (Table 1). These criteria provide the basis for assessing the current status of multi-stakeholder collaboration in a given landscape, and for discussing priorities for the future. After that, platform members can decide upon specific desired outcomes and collaborative action. Desired outcomes can be identified at different spatial levels (e.g., farm, municipality, and landscape) and different temporal scales (short-term, medium-term, and long-term). When the desired outcomes have been agreed upon, platform members can discuss and define indicators that would need to be assessed to measure progress over time (see, e.g., Sayer et al. 2016a, who provide an example of participatory monitoring using the capital assets concept of the sustainable livelihoods framework).

**Table 1:** Looking ahead: Criteria to identify priorities for multi-stakeholder collaboration in a landscape

Aspirations	Criteria
Shared long-term goals and action plan	Stakeholders have shared long-term goals for the landscape
	Stakeholders work together on the basis of a landscape action plan
Practices and policies advance conservation, livelihood and production objectives	Stakeholders work together to promote environmentally friendly production practices and policies
	Stakeholders work together to align conservation practices and policies with livelihood and production objectives
Improved monitoring and land-use planning	Stakeholders jointly monitor developments in the landscape
	Stakeholders catalyze more participatory processes in land-use planning
Responsive institutions	Stakeholders keep each other informed and learn from each other
	Stakeholders use information from other stakeholders to make decisions

#### Looking inward: what is the quality of the multi-stakeholder process within the platform?

The quality of the process within a multi-stakeholder platform is likely to affect the platform’s overall effectiveness. To assess the quality of multi-stakeholder processes we distinguish between two types of process variables: those related to good governance principles and those that can be considered conditions for effective operation of the platform. Good governance principles are widely accepted as guiding principles in development practice (Gisselquist 2012) and are part of the UN Sustainable Development Goals (United Nations 2015). The importance of good governance principles such as participation and transparency is stressed in literature on multi-stakeholder processes (Brouwer et al. 2013) as well as in literature on the governance of natural resources (Lebel et al. 2006; FAO 2011; Carr et al. 2012; USAID 2013; Davis et al. 2013). In the context of landscape approaches, good governance principles relate, amongst others, to participation of weaker actors, so that they can meaningfully engage in discussions (Kozar et al. 2014; Minang et al. 2015). We identified the following three good governance principles as particularly relevant for multi-stakeholder platforms that are part of ILIs:

- Representation: The platform would have to represent the relevant stakeholders in the landscape, which will depend on the objective of the platform and the specific context.
- Participation & equity: Participation is often only perceived in terms of the number of attendants in meetings, and not in terms of who is heard (see, e.g., German and Taye 2008). Participation can have various intensities, from passive listening to active decision-making. A multi-stakeholder platform ideally encourages the active participation of all stakeholders in all discussions and decision-making.
- Accountability & transparency: Accountability within a platform refers to the extent to which members provide information and explain decisions among each other, and the extent to which they can be sanctioned by other members. It requires transparency of information and decision-making.

Next to the good governance principles, we identified key conditions for successful cross-sectoral partnerships. These include access to sufficient capacities and resources, trust between stakeholders, mutual understanding of purpose, and an adequate level of commitment of the participating stakeholders (e.g., Hardy et al. 2003; Drost and Pfisterer 2013; see also Carr et al. 2012). Literature on landscape approaches further stresses the need for adaptive management and good leadership (e.g., Robinson et al. 2012; Sayer et al. 2014; Minang et al. 2015). Based on this, we propose that any assessment of the process quality of a multi-stakeholder platform would need to include a reflection on the following conditions for effective operation of the platform:

- Capacities: A multi-stakeholder platform will need to harbor, or have access to relevant knowledge and skills. Different types of knowledge and skills are needed for the management of the multi-stakeholder platform itself, as well as for the successful development of joint activities, e.g., to promote sustainable practices and markets, collaborative monitoring, and land-use planning.
- Resources: A platform will need to have access to sufficient financial resources to operate effectively, both in the present and in the future.
- Adaptive management: Landscape processes are dynamic and changing circumstances will have to inform decision-making. This means that the management of a multi-stakeholder platform will need to be flexible and adaptive–continuously reflecting on its outcomes and adapting strategies if necessary.
- Leadership: The selection of platform leadership–if required–should be built upon a legitimate and fair process, and it should be accepted and trusted by all platform members. In line with the spirit of the landscape approach, leadership is ideally spread over different sectors and stakeholders.
- Theory of change: Discussions among stakeholders ideally lead to the identification of shared objectives for the future of the landscape. The next step is to develop a clear and agreed-upon theory of change–a strategy to achieve the objectives that all subscribe to. The theory of change will guide action and can be used as the basis for the monitoring and evaluation of medium and long-term outcomes (Sayer et al. 2016b).
- Facilitation and communication: Facilitation implies efficient and effective organization of meetings and other partner collaboration processes, and the planning and mobilization of agreed actions. Moreover, it is critical for information to be widely shared among partners, ensuring that everyone is always up-to-date.
- Trust: A lack of trust among stakeholders will likely result in a lack of transparency and commitment. Ideally, a platform provides a safe ‘space of exchange’ where stakeholders feel comfortable sharing concerns, values and preferences.
- Commitment: In an effective multi-stakeholder platform the individual members will be committed to the platform itself as well as to the agreements made within the platform. Commitment to a multi-stakeholder process also implies a willingness to compromise and jointly identify solutions that reduce trade-offs and maximize synergies between different interests.

Good governance principles and conditions for effective operation are related, and partly overlap. For example, adhering to good governance principles is expected to contribute to building trust and mutual understanding among stakeholders. Table 2 presents the process principles–and corresponding criteria–that we consider fundamentally important for multi-stakeholder platforms in the context of ILIs.

**Table 2:** Looking inward: Criteria to assess the process within a multi-stakeholder platform

Type	Principle	Criteria
Good governance	Representation	The platform represents all relevant stakeholders in the landscape
		Members accept the way in which platform members are selected
	Participation & equity	All members participate and are heard in discussions
		All members can influence decision making within the platform
	Accountability & transparency	Members can hold each other accountable for their actions and decisions
		Information and decision-making is transparent
Conditions for effective operation	Capacities	Platform members have proper knowledge and skills to realize the platform’s objectives
		Platform members have access to diverse sources of information (including local, scientific, technological and legislative knowledge)
	Resources	The platform has sufficient financial resources to operate effectively
		The platform has a viable plan to secure financial resources in the future^a^
	Adaptive management	Platform’s plans can change based on periodic reflection on its functioning
		Members are able to address complaints/suggestions/conflicts within the platform
	Leadership	Members accept and trust the platform’s leadership
		Members accept the selection process of leadership
	Theory of change	Members agree on most of the platform’s objectives for the future of the landscape
		The platform has a clear and agreed-upon strategy to achieve these objectives
	Facilitation and communication	The platform is effective in the organization of meetings and mobilization of agreed actions
		Information is widely shared among members
	Trust	Members feel comfortable sharing information and making agreements
		Members feel welcome, informed and encouraged to contribute
	Commitment	Members are committed to the discussions and the agreements
		Stakeholders are willing to look for compromises

^a^ When the ambition is to evolve into a permanent governance arrangement, the question is whether the platform can continue functioning after start-up funds run out

#### Looking back: to what extent has the platform met its objective?

When a multi-stakeholder platform has been operational for some time, the assessment of its performance can be used for accountability and learning purposes, and it may sometimes be required by financers. Performance measurement of traditional project interventions usually refers to assessing progress toward specified project objectives (Mascia et al. 2014). When a project has specified and clear intended outputs and outcomes, such a standard form of performance measurement will often suffice—the intended outputs and outcomes then function as reference points for the assessment. However, many multi-stakeholder platforms are not organized as projects. They are often long-term collaborative initiatives that catalyze or undertake a series of related projects. Platforms are dynamic and unpredictable settings. Their objectives and means to achieve them may not be specified in great detail at the onset, and objectives may change over time as a result of discussions and negotiations between the members or the influence of financing organizations. In some cases indicators to measure progress are defined (e.g., Sayer et al. 2016a), but often a framework for systematic monitoring and evaluation is lacking. For such settings the outcome harvesting approach is particularly well-suited, having been successfully applied to assess the performance of networks, platforms, and partnerships (Wilson-Grau and Britt 2012; Rassmann et al. 2013; Wilson-Grau 2015, see Pouw et al. 2016 for a comparable method). Following this approach, we define outcomes as the short-term and medium-term changes that are brought about by the institutional entity to be assessed, in this case the multi-stakeholder platform. These changes may be large or small, intended or unintended, and positive or negative. They do not only refer to tangible changes in the landscape, but may also include changes in institutions, practices, policies, attitudes, and relationships. Outcome harvesting starts with identifying all the changes influenced by the platform. After the outcomes have been identified, the method explores who contributed to each change and how. To structure and focus the harvesting, the platform’s objectives (for as far as they are specified) can be used as reference points. When the platform is financed by one or more donors, it may be useful to consider donor objectives as well.

The extent to which a platform is able to influence long-lasting changes in a landscape tends to depend–at least partly–on its connectedness to government institutions that have the legitimacy to make land-use planning decisions, integrate agreements into legal measures, and enforce them. However, it will often be difficult to embed the outcomes of horizontal multi-stakeholder collaboration into the vertical structures of the state, i.e., the formal planning mechanisms (see, e.g., Van Oosten et al. 2014). In many remote rural areas the effectiveness of government institutions is hampered by unclear or overlapping mandates, conflicts of interest, unclear property rights, corruption, and a lack of resources. When reflecting on a platform’s outcomes it is therefore particularly relevant to assess whether the platform has managed to develop constructive relations with government actors, and whether it has succeeded to have the outcomes of the multi-stakeholder negotiations implemented through the formal administrative system.

### Three Tools for Participatory PME Workshops

Each of the three questions introduced above can be addressed in a workshop with key stakeholder representatives, i.e., the individuals who play (or are expected to play) an active role in the platform. Such a workshop can be planned and facilitated by the leadership of the platform, a sponsoring organization or ‘investor’ in the platform, or a boundary or bridging organization that provides technical assistance or capacity development support to a landscape initiative. In some cases a combination of these organizations and interests may decide to form a team to jointly conduct a workshop, or hire consultants to do so. When seen through the lens of conventional project interventions, looking ahead would typically take place at the beginning of the project, looking inward would be part of adaptive management procedures, while looking back would be part of end-of-project evaluation. However, as multi-stakeholder platforms are often long-term collaborative initiatives (possibly involving several projects), we argue for flexibility. Each of the three questions can be relevant at any time, depending on the status and needs of the platform and its members and supporters. We developed a simple tool for each question (see Kusters et al. 2016).

The tools to look ahead and inward both involve two main steps. The first is to assess the current situation, using the criteria provided in Tables 1 and 2. These assessments are conducted on an individual basis, using a scoring card with a five-point Likert scale, comparable to the landscape scorecard developed by the Landscape Measures Resource Center (Cornell University 2016). Basic analysis of the data can be conducted after the scoring exercise (e.g., calculating the mean and standard deviation for each criterion) and presented to the group. This allows for an immediate reflection on the results. The second step involves an in-depth discussion on priorities and associated difficulties, including the identification of practical next steps.

The looking back tool is based on the outcome harvesting approach (Wilson-Grau and Britt 2012) and will typically be used as a complement to other tools used for monitoring and evaluation (e.g., land-cover change maps, evidence on livelihood improvements or agricultural productivity change, or community observation of endangered species). It has a particular value as an integrative tool that actively engages stakeholders representing different perspectives. A complication with applying outcome harvesting to multi-stakeholder platforms is that it may be difficult to distinguish between outcomes of actions by individual members and outcomes of the platform. After the outcomes have been identified it is therefore important to discuss the added value of multi-stakeholder collaboration, i.e., what would have been different if there would have been no interaction between the different stakeholders?After outcomes have been identified, participants are asked about the factors that obstruct the achievement of the platform’s objectives. These can be related to the way the platform is operating, but they can also be external to the platform. After this, the discussion moves on to identifying options to increase the platform’s effectiveness (Kusters et al. 2016).

### Pilots

We tested the method during workshops in Indonesia and Ghana. After each workshop we evaluated the tools with some of the participants and facilitators. Based on the feedback we received, the method was further refined. Below we will present some of the workshop results, and briefly reflect on the experience in each country.

#### Exploring possibilities for multi-stakeholder collaboration in the Juabeso-Bia landscape, Ghana

In June 2016 we organized a workshop in the Juabeso-Bia landscape in Western Region, Ghana. The Juabeso-Bia landscape is made up of a mosaic of cocoa agroforest, cropland and remnant forest, located between the Krokosua Hills Forest Reserve and the Bia National Park. The purpose was to provide stakeholders in the landscape with an opportunity to explore the ways in which they can collaborate in the future (looking ahead). This was meant to serve as input for the planning of the Ghanaian program of the Green Livelihoods Alliance, which is a Netherlands-based program that works with local partners aiming to improve the governance of forested landscapes. The workshop was attended by 45 participants, representing CSOs, NGOs, small-scale miners, local government agencies, park authorities, traditional leadership and members of the Landscape Management Board, representing cocoa farmers in the area.

The participants were first asked to make value judgments about the current state of multi-stakeholder collaboration in the landscape, using a scoring card with the criteria presented in Table 1. On-the-spot analysis showed that the lowest scores were for ‘responsive government institutions’ and ‘improved monitoring and land-use planning’. There appeared to be a widely felt lack of connection between the government and other stakeholders in the landscape. The criterion ‘stakeholders work together to promote environmentally friendly production practices and policies’ scored relatively high. This was explained by ongoing efforts of the Rainforest Alliance, Olam International and the Landscape Management Board to make cocoa production more sustainable and climate-smart. However, there were many worries about production activities outside of the cocoa sector, particularly in mining and logging. A major complaint was that the government failed to consult other stakeholders when giving out concessions to gold miners and sawmills. Permit processes were said to lack transparency and several participants argued that the issuance of permits was often related to the personal interests of government officials.

In the second part of the workshop participants discussed priorities. In general terms participants agreed that it would be desirable to create a broad multi-stakeholder platform to enable discussions and negotiations about the future of the landscape. Specific attention would have to be paid to actively involving women. Participants also agreed that the local government would need to make planning processes more transparent and that local stakeholders should be given practical possibilities to get involved in planning procedures. Lastly, participants stressed the need for better monitoring of developments in the landscape, particularly related to the allocation of permits and compliance with regulations by miners and loggers.

At the end of the day we asked the participants to reflect on the workshop’s usefulness. The overall sentiment was that it had been a first step toward further landscape-wide collaboration. Participants particularly appreciated that the workshop had provided them with a structure for an open conversation, without ideas being imposed on them. The downside, however, was that discussions had remained general, i.e., ideas for collaboration had not been translated into practical next steps. This is partly explained by the high number and diversity of workshop participants, several of whom had no previous exposure to the concepts discussed. This meant that the facilitator had to spend a lot of time explaining the assessment criteria, and there were long discussions about the exact definitions of certain terms (e.g., what is a stakeholder?). The experience highlights that a high number and diversity of workshop participants is likely to contribute to trust-building and the development of general discussions, but may make it more difficult to agree on action points and decisions. More generally, this points to a tension that may exist between the wish to have an inclusive and open platform where members can raise their own issues, and the wish for an institutional mechanism with a clearly defined objective that can effectively realize practical on-the-ground changes.

#### Reflecting on institutionalized multi-stakeholder collaboration in East Kalimantan, Indonesia

In May 2016 we organized a workshop with the members of an existing multi-stakeholder platform in East Kalimantan, Indonesia (as the workshop was internal we decided to keep the platform anonymous). The platform was established by government decree in 2004, and has since had the authority to manage two protection forests. Both protection forests are considered crucial for the provision of water to the neighboring city and its oil industry. The platform consists of a dozen member organizations, including the local government, NGOs, local communities, companies, local media, and research institutions. All the member organizations were represented during the workshop, with the exception of the environmental office of the local government. After a short introduction by the facilitator, participants started with scoring the performance of the platform on the process criteria presented in Table 2. On-the-spot analysis of the scores drew attention to a particularly low score for the criterion ‘all members participate and are heard in discussions’. The ensuing discussion revealed that the platform seldom has all members present at its meetings, with an average attendance of about 50%. This was considered problematic, as decisions are made during meetings without informing the other members. Participants argued that the platform would need to improve information flows by developing a simple communication routine for each meeting–involving teleconferences, text-messaging, and a Facebook group. When discussing the extent to which members agree on a theory of change it became clear that there are different views on the question whether or not community forestry should be allowed within the boundaries of protection forests. Discussions around this issue even resulted in one conservation-oriented NGO member leaving the platform some years earlier. The process assessment also yielded lively discussions on issues related to leadership (e.g., is it desirable to have leadership shared between different sectors?) and transparency (e.g., is there a tension between the wish for full transparency and the role of the platform to enable informal bi-lateral discussions among its members?).

The second part of the workshop focused on the outcomes assessment (looking back). It yielded a long list of achievements, as well as difficulties. Most notably, members indicated that there is an increasing pressure on the protection forests due to planned infrastructural developments around the borders of the protection forests, including housing estates and mining concessions. Also, community representatives stressed that the tenure status of many farms remains unclear, which is leading to tensions between farmers and developers, and among farmers themselves.

The last part of the discussion focused on priorities and next steps. Participants agreed that the platform should prioritize efforts to increase the buffer zone as well as a revision of the spatial plan. The latter would have to include a reconsideration of planned housing and mining developments. As a first step toward clarifying the tenure status of farms the platform proposed mapping all settlements within the protection forest. Also, several members suggested to re-establish contact with the conservation NGO that left the platform, by identifying the long-term shared objectives, while at the same time agreeing to disagree on some matters. The most urgent issue appeared to be related to a new government regulation that moves the authority for the management of forest areas from the municipal and district governments to provincial governments. The platform agreed on establishing a working group that will set up a meeting with the provincial governor about the possibilities to integrate the existing multi-stakeholder platform into the new provincial management structure.

Reflecting on the workshop, participants expressed particular enthusiasm about the process assessment. It had provided them with a structure to discuss matters they would normally avoid discussing because of their potentially sensitive nature (e.g., related to membership, leadership, commitment, and trust). An open discussion about these issues was considered of crucial importance. The outcomes assessment had made clear that ideas about outcomes that exist within a platform do not necessarily tally with non-members’ ideas about the platform’s performance. This highlights the importance of distinguishing between a self-assessment and an external evaluation. Although an external evaluation could make use of a participatory self-assessment workshop, it would also need to include the information and ideas of stakeholders that are not part of the platform.

### Discussion: Toward Effective PME of Multi-Stakeholder Platforms

Discussion and collaboration between stakeholders is widely considered a sine qua non for the inclusive and sustainable governance of landscapes, and conservation and development organizations as well as other actors are therefore likely to continue investing in multi-stakeholder platforms. Realistically, many of the fundamental challenges to effective multi-stakeholder platforms that were outlined in the introduction (such as high transaction cost and power differences) are likely to remain. Within this context, however, the method presented above is expected to help improve the performance of platforms by providing a framework for PME with an emphasis on learning. It means to provide relatively simple tools to structure complex and unpredictable multi-stakeholder processes.

The method’s success largely depends on the active engagement of platform members. A critical condition for this is that the relevant stakeholder representatives are willing and able to participate in a workshop. To have participants committed to the workshop, the purpose and expected outcomes of the exercise should be made explicit. There should be a clear and common understanding about how the results will be used, and of the potential follow-up measures, i.e., the ability to act on recommendations that may arise from the assessment (cf. Guijt and Woodhill 2002). To help generate commitment to the process it is important to ensure that participants understand how much their knowledge and participation is valued and appreciated by the organizers of the workshop. This can be demonstrated in small and meaningful ways such as enabling them to co-design parts of the workshop process, creating a cordial atmosphere, and covering transportation and other logistical costs to participate.

Another condition for a successful PME workshop relates to the ability and willingness of participants to reflect critically on themselves and others within the platform. The extent to which they will be willing and able to do so will depend on cultural norms, conflicts and tensions that may exist, and the potential of power asymmetries within the group. Sometimes time and skilled facilitation will be needed to create an atmosphere of trust and openness, and to overcome barriers for people to speak freely. An important role for the facilitator in this regard is to convey the idea that critical reflection will help to identify opportunities for improvement. This means that the discussion should focus not only on success, but also on past failures and difficulties. The recognition of failures should be rewarded, in order to learn from them (see, e.g., Clark et al. 2016). Likewise, there should be sufficient attention for the unexpected or unintended consequences; developments that had not been foreseen, but which influence the functioning of the platform.

Although the method is specifically designed for participatory PME purposes, it may also provide a framework that can be used for scientific inquiry. For example, using parts of the method to collect data for a large number of platforms may help to shed light on assumptions that underlie investments in multi-stakeholder platforms in the context of ILIs. These may include, for example, the expectation that multi-stakeholder platforms allow for meaningful and equitable stakeholder participation, as well as the idea that the results of a multiple stakeholder process will reflect a balance between conservation, livelihood, and production goals. However, for scientifically robust comparative analyses of outcomes and the relationships between processes and outcomes, more rigorous and standardized ways to document outcomes will probably be necessary.

As yet, there is no robust scientific evidence concerning the effectiveness of multi-stakeholder platforms in achieving conservation, livelihood, and production goals in the landscape. Still, investments in such arrangements as part of ILIs are likely to continue and there is a need for methods to aid their planning and to critically assess their processes and outcomes. The method presented in this article can be used as a starting point. It is catered to the specific challenges of multi-stakeholder platforms in ILIs and has been developed with the intention to do full justice to the knowledge and experience existing within these platforms.
